# Implication of the VRK1 chromatin kinase in the signaling responses to DNA damage: a therapeutic target?

**DOI:** 10.1007/s00018-018-2811-2

**Published:** 2018-04-20

**Authors:** Ignacio Campillo-Marcos, Pedro A. Lazo

**Affiliations:** 1 Experimental Therapeutics and Translational Oncology Program, Instituto de Biología Molecular y Celular del Cáncer, CSIC-Universidad de Salamanca, 37007 Salamanca, Spain; 2 Instituto de Investigación Biomédica de Salamanca (IBSAL), Hospital Universitario de Salamanca, 37007 Salamanca, Spain

**Keywords:** VRK1, H2AX, NBS1, 53BP1, p53, Phosphorylation, DNA damage response, Ionizing radiation

## Abstract

DNA damage causes a local distortion of chromatin that triggers the sequential processes that participate in specific DNA repair mechanisms. This initiation of the repair response requires the involvement of a protein whose activity can be regulated by histones. Kinases are candidates to regulate and coordinate the connection between a locally altered chromatin and the response initiating signals that lead to identification of the type of lesion and the sequential steps required in specific DNA damage responses (DDR). This initiating kinase must be located in chromatin, and be activated independently of the type of DNA damage. We review the contribution of the Ser-Thr vaccinia-related kinase 1 (VRK1) chromatin kinase as a new player in the signaling of DNA damage responses, at chromatin and cellular levels, and its potential as a new therapeutic target in oncology. VRK1 is involved in the regulation of histone modifications, such as histone phosphorylation and acetylation, and in the formation of γH2AX, NBS1 and 53BP1 foci induced in DDR. Induction of DNA damage by chemotherapy or radiation is a mainstay of cancer treatment. Therefore, novel treatments can be targeted to proteins implicated in the regulation of DDR, rather than by directly causing DNA damage.

## Introduction

### Genome stability

Genome stability is essential for the maintenance of species, but, at the same time, genetic variation is necessary for their evolution. Therefore, in all species, there are several mechanisms aiming to protect DNA from genetic damage of endogenous or exogenous origin. Endogenous DNA damage is a consequence of the biological properties of cells, and includes oxidative stress, replication errors, transcriptional errors, or metabolism of DNA, to which cells are continuously exposed [1]. Alternatively, exogenous factors such as ultraviolet light, ionizing radiation, or chemicals also cause DNA damage to which exposure is frequently transient. The DNA damage has many different forms, single- or double-strand breaks, nucleotide, or base modification [1]. To cope with all of them, cells have developed several specific DNA repair mechanisms, which increase their complexity in higher organisms because of the chromatin organization. Double-strand breaks constitute the most serious form of DNA damage that has two alternative repair mechanisms depending of the situation of the cell cycle. During replication, DNA double-strand breaks (DSBs) are repaired by homologous recombination (HR) using as template the other chromatid. In non-dividing-cells or in G0/G1 phases, DSBs are repaired by non-homologous end-joining (NHEJ) [2].

Independently of the origin of DNA lesions, these lesions have to be detected rapidly and efficiently before cells divide, to avoid transmitting the damage to their progeny. Because nuclear kinases are capable of rapidly and reversibly responding to changes in the cell and its environment, and of integrating diverse stimuli, they are likely to be involved in sensing, triggering, regulating, and organizing the sequential steps that are needed for a correct and specific DNA damage response.

Cells are continuously exposed to DNA damage and it can occur at any time during the cellular lifetime. The number of normal cell division is limited to approximately 40 because of telomere shortening, which implies that, in the life of the organism, most cells are not dividing at the time of exposure to DNA damage [3, 4]. Furthermore, cells are most of their lifetime in the G0/G1 phases, in which homologous recombination is not functional [5], but are exposed to DNA damage. Furthermore, stem cells have an enhanced response to DNA damage mediated by the NHEJ pathway [6]. Therefore, most of the DNA lesions will occur and have to be repaired in the absence of replication. Very often, there is a large time interval between the moment in which DNA damage occurs and the time when an individual cell replicates, in which most cells are non-dividing, and are thus able to pass the mutation to their daughter cells. Consequently, each cell has to deal individually with this problem and to respond independently of its particular situation, which is very variable within a tissue. Cells are either resting or dividing, and their individual position within a tissue implies that cellular interactions are heterogeneous depending on its location. DNA repair mechanisms have to function in all these different cellular contexts. In the particular case of neurons, by their exposition to oxidative stress, the accumulation of DNA damage might be a pathogenic mechanism for deterioration of neurological functions associated with aging. Recent evidence indicates that a significant proportion of the DNA damage is of endogenous origin [7, 8]. Francis Crick predicted that several redundant mechanisms must exist to repair damaged DNA and maintain genome integrity [9]. Since then, several pathways have been identified [10–13]. Induction of DNA damage is a mainstay of cancer treatment, and the specific targeting of regulatory proteins implicated in DDR can lead to the development of new drugs.

### Chromatin and DNA damage

The cellular response to DNA damage has to be initiated and triggered at the site of the DNA lesion, independent of its type. DNA damage causes a local distortion of the double helix, and of its associated nucleosomes, which is reflected in the local chromatin organization. Chromatin can function as a signaling platform that has effects not only on its remodeling, but can also send signals to other processes involved in nuclear dynamics [14]. When cells encounter a stress such as DNA damage, the activation of complex signaling networks triggers the detection and repair of the damage in a specific and sequential process, before returning to the homeostatic equilibrium. These networks integrate a wide variety of signals from inside the cell, transduced through protein kinases [10–12], to ultimately control cell cycle arrest or progression in the case of dividing cells [15]. Moreover, the chromatin-signaling platform regulates DDR, cell cycle checkpoints, cell death, and senescence, among others. All these processes are associated with the maintenance of genetic stability and the transmission of a mutation-free genome to daughter cells. The major pathological consequence of DNA damage is the potential transmission of mutations to their progeny [16], which are implicated in aging and cancer [17]. In addition to the role of DNA damage in cancer, alterations in DNA repair genes are also associated with neurodegenerative diseases [18], since neurons are not dividing in most of the individual lifetime and have to repair these DNA lesions. Most research into DNA damage responses has been studied in the context of replication and cell division [16].

In the highly organized eukaryotic chromatin, the most vulnerable DNA is the fraction that is transcriptionally active at the time of exposure to damaging agents, particularly in resting or non-dividing cells, such as stem cells or neurons. In these locations, DNA has to relax and open to allow the access of RNA polymerase and permit gene transcription. In these transcriptionally active regions, DNA is more exposed and vulnerable, particularly in non-dividing or cells in G0/G1. Therefore, in an individual resting cell, the response to DNA damage does not have to be linked to cell division, differentiation state, or the cell location and its interactions within a tissue. Even in dividing cells, the G1 phase last several hours before entering replication. DNA damage has to be detected, identified, and repaired immediately in all different types of situations.

### Cellular response to DNA damage

The cellular reaction to DNA damage involves two major aims; one is to protect the DNA, and the other to protect cells and the organisms from the consequences of unrepaired DNA damage. The cellular protection against DNA damage is mediated by arresting cell cycle in proliferating cells, so that damage can be repaired before its transmission to daughter cells. However, if DNA damage is excessive and cannot be repaired, the alternative response is mediated by the induction of cell death, and in that way, there is no progeny of mutated cells. These two types of responses are associated with p53 and activated by different types of DNA damage.

DNA repair requires a sequential reorganization of chromatin to allow for the different and consecutive steps in each repair pathway, which includes protection of damaged DNA, recognition of the type of lesion, recruitment of specific repair mechanisms, ligation of DNA ends, and restoration to its normal chromatin organization. After DNA damage, in addition to the DNA lesion, the initial effect is a local distortion of chromatin, which is the initiating event to trigger the cascade of DNA repair processes. As organisms increased in their complexity, new regulatory elements are necessary not only to coordinate different functions in DDR, but also to adjust to their much more complex and dynamic structure of chromatin. Therefore, new regulatory mechanisms that integrate and coordinate basic processes are necessary. In this context, new regulatory elements have evolved from preexisting proteins. A candidate for this role must be a chromatin protein with a reversible enzymatic activity. Among the 518 kinase of the human kinome, vaccinia-related kinase-1 (VRK1) is a potential candidate for this role because of its association to chromatin and its targets, with the exception of chromosomes condensed in mitosis [19, 20].

## VRK1 roles in chromatin

### The VRK1 chromatin kinase

VRK1 is a Ser-Thr kinase that belongs to the VRK family that diverged early from branch of the human kinome that led the casein kinase family [21]. Bacteria and yeast have no VRK or p53 members, invertebrates such as *D. melanogaster* or *C. elegans* have one member, and mammals have three members in their respective families. The complexity of VRK family [22] parallels that of p53 [23] and the autophagic DRAM (death-related autophagic modulator) [24]. This increased complexity during evolution is likely to reflect the need for additional regulatory or coordinating roles as organisms and their functions became more complex. In mammals and *C. elegans,* it is known as *Vrk*-*1* [25], and in *D. melanogaster* as nucleosomal histone kinase 1 (NHK-1) [26].

**Fig. 1:**
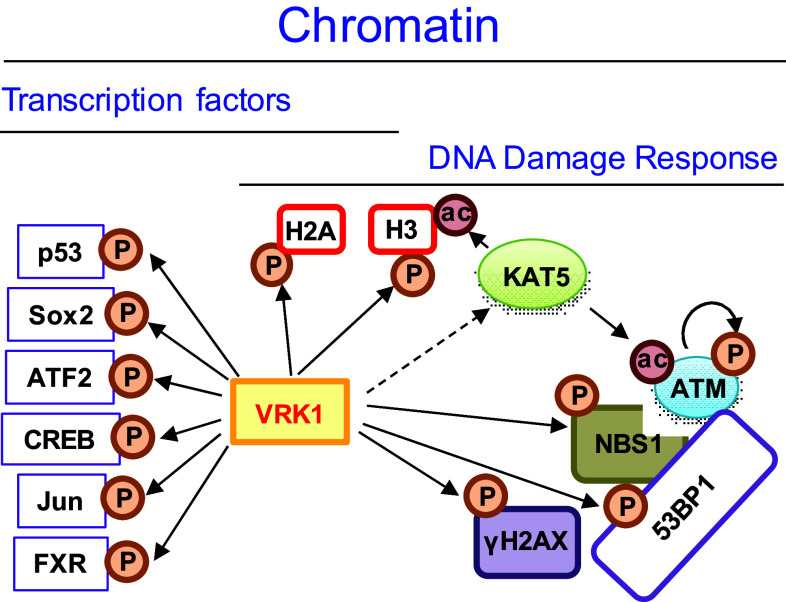
VRK1 relation with transcription factors and DNA damage response proteins in chromatin

VRK1 is a Ser-Thr kinase in nuclei [19] that is located on chromatin in resting cells and in all phases of the cell cycle covering all DNA, except when chromosomes are already condensed in mitosis [27, 28], in which VRK1 is ejected from mitotic chromosomes. When chromosomes segregate, VRK1 returns to chromatin in daughter cells. VRK1 forms stable complexes with several different types of chromatin proteins, ranging from histones, transcription factors, and proteins involved in DNA repair processes (Fig. 1). The proteins more closely associated with DNA are histones [29], which are organized in nucleosomes and in direct contact with DNA, contributing to chromatin spatial organization. VRK1 is detected in the chromatin fraction forming a stable complex with histone H3 [29]. Moreover, VRK1 phosphorylates histones H3 [27, 29, 30], H2A [31, 32] and H2AX [29]. Therefore, it is very likely that nucleosome organization can be modified by covalent modifications because of histone phosphorylations by VRK1. This regulation of histone covalent modifications is essential for different functions, normal or pathological, requiring a dynamic chromatin reorganization [33, 34].

An additional role of VRK1 as a chromatin kinase is its association with transcriptional complexes, where it interacts and phosphorylates several transcription factors that include p53 [28], CREB [35], ATF2 [36], c-Jun [37], Sox2 [38], and the farnesoid X receptor (FXR) [39].

### VRK1 as a sensor of chromatin alterations

Chromatin in interphase has a very large size and DNA lesions can occur at any place, heterochromatin and euchromatin, which are likely to have a different sensitivity to DNA damage. Alterations of DNA by strand breaks or chemical modifications, such as oxidation, alkylation, or intercalation among others, will alter the chromatin organization by introducing a local distortion [40, 41], which is a likely initiating event for triggering the complex processes of DNA repair [42–44]. However, responding to DNA damage requires the coupling of chromatin distortion to a signal transduction system, probably mediated by a nuclear chromatin kinase.

A requirement for a sensor kinase is that its activation is independent of the type of DNA damage and, therefore, is not associated to any particular type of DNA damage. In this latter case, the kinase involved will participate in specific steps of a particular DNA damage, as is the case for ATM, ATR, or DNA-PK in the response to double-strand DNA breaks [45]. In the particular case of VRK1, its kinase activity increases tenfold after induction of DNA damage independently of its type, which includes pyrimidine dimers caused by ultraviolet light, single-strand DNA breaks caused by hydroxyurea treatment, or double-strand DNA breaks induced by either doxorubicin or ionizing radiation [46].

Early sensor mechanism of DNA damage must fulfill some basic requirements, be a nuclear enzyme that interacts with basic chromatin components in nucleosomes, and be a capable of an immediate signaling reaction that is also reversible. In this context, a kinase, such as VRK1, is a very suitable candidate for this role [29, 46, 47].

Other important early proteins at the site of specific types of DNA damage are Ku70/Ku80 (XRCC6/XRCC5), which have to re-localize and interact with free DNA ends at the breakpoints, mainly in double-strand breaks [48], a subtype of DNA damage, or in telomeres [48, 49]. It is unknown whether these proteins are targets of VRK1, but it is a real possibility. Ku70 and Artemis have multiple phosphorylation sites, but the kinases involved in their specific phosphorylation and their regulation are unknown. Telomeres are naturally occurring DNA ends in chromosomes and there is evidence for a role of VRK1 in their maintenance [50]. Moreover, VRK1 phosphorylates hnRNP A1 (heterogeneous nuclear ribonucleoprotein A1) and facilitates its binding to telomeric ssDNA and telomeric RNA [50].

### VRK1, chromatin relaxation, and histone acetylation

DNA damage and local disruption of chromatin are associated with an increase in histone acetylation, which is mediated by KAT (lysine acetyl transferase) proteins. Histone acetylation extends over an area of several hundred kilobases flanking the damaged DNA site [51, 52], which requires the local activation of KATs by a not yet identified mechanism. Defects in histone acetylation are associated with an increase in cellular sensitivity to DNA damage as a consequence of a defective DNA repair [53, 54]. Furthermore, acetylation of histone H4 in Lys16 disrupts the interaction between H4 and H2A–H2B, and facilitates the relaxation of chromatin [55, 56]. Consistently, the inactivation of KAT5/Tip60 blocks the opening of chromatin at DSBs (double-strand breaks) that are required to facilitate the repair process [52]. Induction of DNA damage by UV light or radiation causes an increase in histone acetylation [52, 57]. Depletion of VRK1, a nucleosomal chromatin kinase, causes a loss of histones H3 and H4 acetylation, which are necessary for chromatin relaxation, either in basal conditions or after DNA damage, independently of ATM and p53 [29]. VRK1 knockdown also causes a loss of specific histone acetylations, including H4K16 acetylation (H4K16ac), induced by DNA damage [29]. ATM-null cells, such as the HT144 cell line, has a high endogenous level of H4K16ac that is also lost by depletion of VRK1 [29]. In ATM^+/+^ cells, this acetylation induced by IR does not occur in the absence of VRK1 [29]. These results indicate that VRK1 is a good candidate to regulate the enzymes involved in epigenetic modifications of chromatin. DNA damage causes a local distortion of chromatin that can affect its different covalent modifications. Consequently, the regulation and coordination of histone modifiers such as acetylases, deacetylases, methylases, and demethylases is very poorly understood. Moreover, VRK1 also directly phosphorylates histone H2A in T120 [32], which is next to K119 ubiquitinated, and both modifications are functional alternatives, being T120 phosphorylation an activator of chromatin. Thus, two histones in nucleosomes, H3 and H2A, are directly regulated by VRK1. Furthermore, histone H4 is not a phosphorylation target of VRK1, but its covalent modification by acetylation is sensitive to VRK1 in an ATM-independent manner, since it is detected in ATM-null cells [29].

It is important to remark that the sensor kinase activity has to be regulated by protein–protein interactions. In this context, the C-terminal region of VRK1 has a low complexity structure, which is very flexible and can adopt different conformations [58]. This C-terminal region can fold and block the active site of the kinase [58] and proteins interacting with this region can modulate the activity of VRK1. Two proteins that inhibit the VRK1 kinase activity have been identified, macrohistone H2A1.2 in interphase [59], and Ran-GDP, but not Ran-GTP [60], which have an asymmetric nuclear distribution [61].

## VRK1 in DNA damage responses

### VRK1 and histone H2AX

VRK1 directly and stably interacts with histones H2AX and H3 in basal conditions, and is able to phosphorylate them in vitro with purified proteins in Ser139 and Thr3, respectively [29]. The early response to DNA damage requires the phosphorylation of H2AX in Ser139 (γH2AX). γH2AX covers large areas of DNA surrounding the site of DNA damage [62] and protects DNA from exonuclease attack. This γH2AX organization can also function as a platform for the recruitment of proteins that participate in sequential DDR steps, such as NBS1, 53BP1, or BRCA1, among others [40, 63]. Phosphorylation of histone H2AX in Ser139 (γH2AX) is a mark of an early reaction to DNA damage that can be detected by formation of γH2AX foci [62, 64]. The phosphorylation of H2AX and the formation of γH2AX foci induced by ionizing radiation (IR) are lost by depletion of VRK1 and can be rescued by kinase-active, but not by kinase-dead, VRK1 [29]. This effect of VRK1 is also independent of ATM, suggesting that VRK1 is an upstream participant. VRK1 is also necessary for the activation of ATM and CHK2 in response to IR [46]. However, in the absence of ATM, the γH2AX foci induced by IR have a smaller size, which indicates that both kinases cooperate either in the formation or stabilization of the foci [29]. This latter possibility might be a consequence of the effect of VRK1 on the stability of NBS1 [47]. In this context, VRK1 is a novel chromatin component that reacts to its alterations and participates very early in DDR by itself and in cooperation with ATM [29].

### VRK1 and specific DNA damage response proteins

Because of the physical association of VRK1 with chromatin, VRK1 has also been implicated in the regulation of DDR proteins. The VRK1 kinase has also been directly associated with different components in DDR pathways, which have been studied in the context of the response to DSBs, in both resting and cycling cells as well as in ATM-null and p53-null cells. VRK1 physically interacts and directly phosphorylates specific proteins participating at different sequential stages of DDR, which include H2AX [29], NBS1 [47], and 53BP1 [46, 65] in NHEJ [66, 67]; and all of these activating phosphorylations are lost by VRK1 depletion. Intermediate steps in DDR signal transmission are well known. The most common pathways in DNA damage response (DDR) implicate protein phosphorylation by different kinases such as ATM [10], ATR [11], and DNA-PK [12]; all members of the PI-3K family, which have been mostly studied in the context of cell division and cell cycle checkpoints [15]. In response to double-strand breaks induced by ionizing radiation (IR), the 53BP1 scaffold protein is recruited to IR-induced foci (IRIF), and is an important marker for monitoring cellular DDR by NHEJ. 53BP1 foci induced by ionizing radiation or doxorubicin are intermediate steps in DDR activation [68, 69] and are known to be regulated by ATM in response to DSBs [70], and by ATR in response to replication stress [71]. However, it is also known that DNA damage response can be ATM-independent [72]. The effect of VRK1 in DDR is insensitive to inhibitors of PI3KK proteins that target ATM and DNA-PK [46]. This suggests that there are alternative kinases participating in DDR induced by ionizing radiation. The complete molecular components that sequentially participate in DDR, particularly regulatory proteins, remain unknown. In this context, VRK1 knockdown also prevents the activating phosphorylations of ATM in Ser1981, CHK2 in Thr68, and DNA-PK in Ser2056, all induced in response to IR [46], suggesting that VRK1 is an early and upstream component in this DDR process.

### VRK1 and NBS1 in early DDR

Cellular responses to DNA damage require the formation of protein complexes in a highly organized fashion. In resting cells, VRK1 plays an important role in the formation of ionizing radiation-induced foci formed by γH2AX, NBS1, and 53BP1 during DDR. The MRE11 complex holds together the two free ends of the broken DNA. This complex, formed NBS1–Mre11–Rad50, is highly dynamic and has a very complex organization [66]. Phosphorylation of NBS1/nibrin is necessary for the recruitment of ATM to damaged sites and for the stabilization of the repair complex [73]. VRK1 is activated by DNA double-strand breaks induced by ionizing radiation (IR) or doxorubicin, and specifically phosphorylates NBS1 in Ser343 [47] and 53BP1 in serum-starved cells and ATM-null and p53-null cells [46], indicating that they are independent of both p53 and ATM activation [47], and consistent with VRK1 role as an early step in the response to DNA damage. Depletion of VRK1 causes a loss of NBS1 stability that is prevented by treatment with the MG132 proteasome inhibitor [47]. This phosphorylation mediated by VRK1 protects the NBS1 protein of RNF8-mediated ubiquitination [47]. Therefore, it is likely that NBS1 phosphorylation by VRK1 contributes to the stabilization of foci, and facilitates the recruitment of additional participants in the specific DNA repair process, such as kinases of the PI3K family, ATM, ATR, or DNA-PK, for specific signaling steps or pathways in DDR [45].

### VRK1 and 53BP1 in NHEJ

Double-strand breaks are the most serious form of DNA damage, particularly in cells that are resting or in the early phases of the cell cycle, which includes differentiated resting cells, as neurons, and stem cells. Under these conditions, these DSBs are repaired by non-homologous end-joining (NHEJ); and one of its main components is 53BP1, a scaffold protein that forms foci induced by DNA damage [74]. VRK1 stably interacts with 53BP1 in the region comprised between residues 955-1354, which is implicated in the interaction with H2AX, but its phosphorylation site is in Ser25/29 within the 53BP1N-terminal region and occurs even in the absence of ATM (null cells) [46]. VRK1 depletion causes a defective formation of 53BP1 foci induced by ionizing radiation or doxorubicin, both in number and size, which requires a kinase-active VRK1 protein for their rescue [46]. Moreover, this effect of VRK1 on 53BP1 foci is insensitive to ATM and DNA-PK inhibitors and is functional in p53-null and ATM-null cells. All these data indicate that the effect of VRK1 is independent of both ATM and p53 [46], and that VRK1 activation in response to DNA damage is a novel participant in the NHEJ mechanism of mammalian DNA damage responses [29, 46, 47].

## Cellular protection mediated by VRK1 and its target p53

### VRK1 forms a complex and phosphorylates p53

**Fig. 2:**
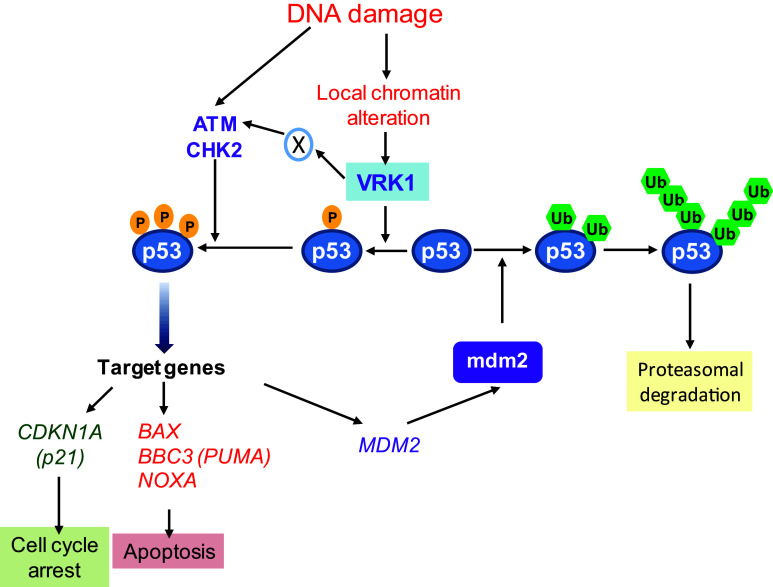
Kinase activation induced by DNA damage and the regulation of p53 in G0/G1 cells. An enzyme (X) activated by VRK1, and that has not yet been identified, mediates the activation of ATM

The p53-mediated responses induced by DNA damage have two major roles in the context of cellular protection (Fig. 2). The first one is preventing the transmission of damaged DNA to daughter cells during cell proliferation. This p53 action is mediated by the induction of a cell cycle arrest, and forms part of cell cycle checkpoints [75, 76]. The other role is the protection of the organism from the consequences of accumulating cells with damaged DNA, which is mediated by induction of cell death [77]. The p53 transcription factor mediates these two main protective responses that are regulated by p53 immediate phosphorylation in response to DNA damage. These cellular responses have a different temporal order because of the covalent modifications, which are immediate as the stabilization of p53, or require hours, such as the induction of specific gene expression.

The stabilization and activation of p53 is performed by several Ser-Thr kinases that target different residues within the p53N-terminal transactivation domain (Fig. 2), and have different sequential roles [78–80]. In the absence of stress or DNA damage, the basal intracellular level of the p53 protein is very low, but it is always present. This basal low level of p53 is necessary to initiate a fast response to cellular stress by its immediate phosphorylation. The p53 phosphorylation in several residues within its TAD1 region (residues 1–46) is the main determinants of the stress response [79]. To trigger an immediate reaction to DNA damage, the response will be greatly facilitated by the formation of a stable and inactive complex between p53 and one of its regulatory kinases that are activated by DNA damage. In non-damaged cells, the basal low p53 level is partially forming a stable complex with VRK1, which are detected by reciprocal immunoprecipitations, and are detected in resting and cycling cells [81]. This basal VRK1-p53 complex forms a basic early warning system for detection of cellular stress and its activation is induced by DNA damage caused by ultraviolet light, ionizing radiation, or doxorubicin treatments. All these types of DNA damage activate the kinase activity of VRK1, a previous step required for the specific phosphorylation of p53 at Thr18 [28, 81]. Therefore, the subpopulation of basal p53 that is forming a complex with VRK1 facilitates a readiness state of p53 to initiate an immediate activating response in different cellular stress situations [28, 81]. Furthermore, the p53 protein also indirectly plays an important role in epigenetic regulation of chromatin [82].

### Phosphorylation of p53 by VRK1 prevents the interaction with MDM2 and regulates the switch between ubiquitination and transcription

Non-phosphorylated p53 binds to the human MDM2 (HDM2) ubiquitin ligase [83]. VRK1 uniquely and specifically phosphorylates p53 in Thr-18 [28, 84, 85]. This Thr18 residue is critical to maintain the folding of the p53 α-helix required for its binding to a hydrophobic pocket in MDM2 [86]. The phosphorylation of p53 in Thr18 alters the alignment of hydrophobic residues in this α-helix, and this altered conformation permits the p53 binding to transcriptional cofactors. Moreover, this phosphorylation of Thr18 determines the change in binding mode from ubiquitin ligases to transcription factors, and additional p53 phosphorylation in Ser15 or Ser20 [87] contributes to the selection of specific transcriptional cofactors [88]. The specific phosphorylation of p53 in Thr18 places VRK1 upstream of additional phosphorylation in Ser15 and Ser20 mediated by other kinases [79]. The activated ATM-CHK2, ATR-CHK1, or DNA-PK pathways mediate the phosphorylation p53 in Ser15 or Ser20 [78, 79], and all of them are necessary to achieve the full transcriptional activation of p53 [88]. These additional p53 phosphorylations, and their combination, select transcriptional cofactors and activate p53-dependent genes such as *CDKN1A* (p21) expression [89], which induces a cell cycle arrest and senescence [90], and *BAX* that facilitates apoptosis [91, 92], among others. The role of p53 in transcription in these processes has been extensively reviewed [93].

The kinase activity of all these p53 kinases, VRK1, ATM, ATR, and DNA-PK, are inducible by DNA damage, but their spatial organization, coordination, and sequential activation require further studies for its complete understanding. In this context, because of its interactions with histones, VRK1 is a new component that participates very early in the response mechanisms to DNA damage, as well as in specific steps of DDR.

### Activated p53 induces the downregulation of VRK1

Once DNA damage has been repaired, the cell cycle arrest induced by activated p53 has to be reverted. Otherwise, p53 will maintain the cell cycle arrest or even induce apoptosis. This reversal requires the deactivation of p53, which is mediated by its dephosphorylation and subsequent interaction with MDM2. However, the phosphorylation of p53 in Thr18 by VRK1 blocks its interaction with MDM2 and other phosphorylations, in Ser15 and Ser20 further interfere with the interaction [88]. All these phosphorylations have to be removed, to revert the p53-mediated responses, such as a cell cycle arrest, in viable cells. This is accomplished by the regulation by p53 of different target genes that range from ubiquitin ligases, phosphatases, to autophagic proteins [94]. The reversion of activated p53 also requires downregulation of p53 activating kinases, including VRK1 and ATM, so that dephosphorylated p53 is not re-phosphorylated and becomes accessible to MDM2. In this context, p53 induces of the expression of DUSP6 and WIP1 phosphatases targeting ATM [95–97], and that of the DUSP4 phosphatase targeting VRK1 [98, 99]. However, downregulation of p53 activation is more complex and also requires additional deactivation of other kinases, which are mediated by phosphatases, and deacetylation of p53 [94].

The stabilization and accumulation of p53 by VRK1 in response to DNA damage is reverted by a novel p53-dependent activation of autophagy that removes its activating VRK1 [100], a p53 stabilizer, and thus permits p53 dephosphorylation and its downregulation by MDM2 [85, 100, 101]. Among the degradation processes regulated by p53 is autophagy. In normal cells, autophagy contributes to regulate basal levels of cytosolic and particulate proteins [102], a process that is further activated in response to several types of stress, including DNA damage. Autophagy is required for recycling of proteins implicated in negative cell cycle regulation, and can provide a survival strategy to tumor cells [103]. By this process, regulated by p53, cells remove and digest endogenous proteins, particularly those that are very stable, functioning as an important mechanism for tissue remodeling [103] and maintenance of cellular homeostasis [104], but it can also result in a form of cell death, thus having a dual role [105, 106].

**Fig. 3:**
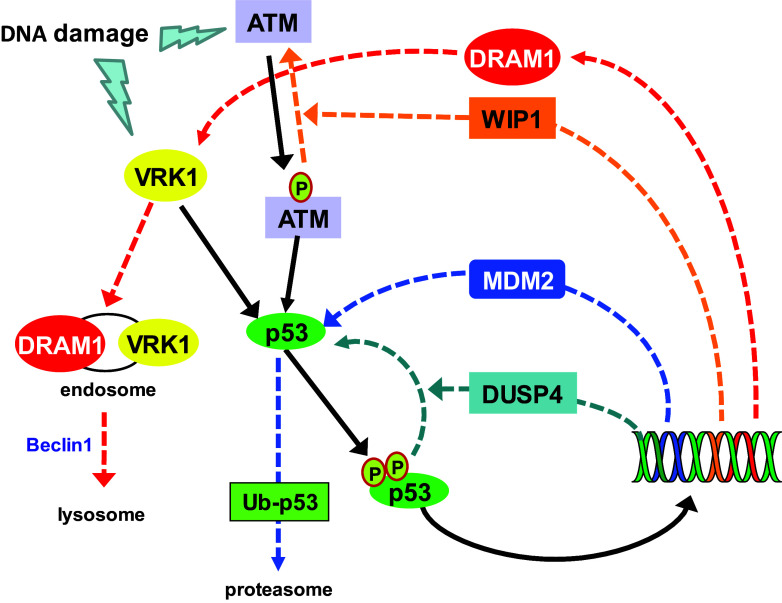
Downregulation of VRK1 by DRAM1 in the autophagic pathway induced by p53 and deactivation of p53 by phosphatases and ubiquitin ligases the proteasome in DNA damage response. Solid black lines represent the activation route. Dashed lines represent the downregulatory routes and each color represents a different route. Kinases: VRK1 and ATM. Phosphatases: WIP1 (wild-type P53-induced phosphatase 1) and DUSP4 (dual specificity phosphatase 4). DRAM1: damage-regulated autophagy modulator 1

The downregulation of VRK1 is a late response that is also mediated by the p53-dependent transcription of *DRAM* (death-related autophagic modulator) [107]. DRAM is a small hydrophobic protein located in the membrane of autophagosomes [108]. Expression of DRAM facilitates degradation of VRK1 in the lysosome, and the elimination of DRAM or Beclin1 prevents the downregulation of VRK1 by proteolytic degradation [85, 100] (Fig. 3). This degradation of VRK1 takes place in the cytosol and is sensitive to the inhibition of nuclear export with leptomycin B and to lysosomal inhibitors [100]. DRAM expression induced by p53 regulates the degradation of stable proteins. DRAM is a novel component of the cell autophagic response [107]. Autophagy is partly regulated by p53-induced *DRAM* expression [107], and p53-induced VRK1 degradation requires entry in the endosomal–lysosomal pathway [85]. In this way, DRAM downregulates VRK1 forming an autoregulatory loop [101] (Fig. 3). Moreover, this autophagic downregulation of VRK1 is altered in tumors with p53 mutations that affect its DNA-binding domain, including all the most frequent mutations detected in human cancer [85, 109], because they disrupt this autoregulatory loop. Consequently, tumors harboring p53 mutations also have very high levels of VRK1, as it has been shown in head and neck squamous cell carcinomas [110] and lung cancer [109], which can also facilitate cell proliferation.

In conclusion, the main mechanism of downregulation of p53 is mediated MDM2, but for this to occur, it is necessary to previously dephosphorylate p53 and its activating kinases, all of which are regulated by p53 [94]. Once p53 is dephosphorylated, it becomes available for its ubiquitination by MDM2 and degradation in the proteasome, which has been extensively reviewed and has become a target for therapeutic intervention with drugs that interfere with the p53-MDM2 interaction, such as nutlins [111].

## Implications of VRK1 in cancer biology

The functions of VRK1 suggest that it is likely to actively participate in tumor biology. Knockdown experiments indicate that VRK1 plays a major role in cell cycle progression and proliferation [27, 112, 113]. Moreover, *VRK1* elimination by CRISPR/Cas9 identifies wild-type VRK1 as an overexpressed oncogenic driver gene [114], consistent with its role in lung adenocarcinomas [115]. In most cell types, the human *VRK1* gene is expressed at different levels and is not mutated in cancer, and it is overexpressed in many cancer types of different origins correlating with a poorer prognosis in breast [116, 117], lung [115], liver [118], glioblastoma [119], head and neck [110], and esophageal cancer [120]. Some driver genes are oncogenic in situations in which they are overexpressed by different mechanisms, as it occurs with members of the *MYC* and *EGFR* families that promote cell proliferation. In the context of tumor growth, the human VRK1 protein has been implicated in the regulation of proliferation and cell cycle progression, where it plays several roles [121]. VRK1 is required for G0 exit, behaving like an early gene such as *MYC* and *FOS,* which facilitate the progression in G1 and passing the restriction point [113]. In this context, depletion of VRK1 prevents the expression of *CCND1* (cyclin D1), since VRK1 directly binds to the human *CCND1* promoter [32], and consequently, retinoblastoma cannot be phosphorylated [110]. Later, in cell cycle progression, VRK1 is also required for the phosphorylation of histone H3 that facilitates the initiation of chromatin compaction in G2/M [27] and cooperates with AURKB in the sequential remodeling of chromatin in the progression of mitosis [122].

However, based on the biological actions of VRK1, either in cell proliferation or DNA damage responses indicates that depending on the cellular context, VRK1 might function as an oncogene or a tumor suppressor or predisposition gene. VRK1 might behave an oncogene because of its roles in the promotion of cell cycle progression and proliferation. However, in other contexts, VRK1 might behave as a tumor suppressor or a tumor susceptibility gene represented by the effects mediated by p53 and those associated with genome stability. These properties, in the context of cancer, can contribute to a poorer prognosis of tumors overexpressing VRK1 because of its contribution to the promotion of cell proliferation and resistance to treatments based on DNA damage.

Due to the essential role played by VRK1, attempts to generate knock-out mice have been unsuccessful. However, the consequences of VRK1 deficiency in animal models have been studied in gene-trap mice with a fifteen percent residual level of VRK1 [123–125]. In this model, deficient animals were sterile, both male and female [123–125], preventing additional studies. The role that VRK1 plays in response to DNA damage in this model was not studied. In one of the studies, the problem was identified as lagging chromosomes during meiosis leading to sterility [124], an observation consistent with the role of VRK1 in dynamic chromatin reorganization. In addition, VRK1 regulates the attachment of chromatin to the nuclear envelop that is mediated by the phosphorylation of BANF1 [126]. The disruption of this process can also lead to alterations of chromatin reorganization in mitosis and affect cell viability [127].

## VRK1 potential as therapeutic target in oncology

Protein kinases, because of their structural characteristics, are candidates for development of inhibitors. Knockdown screening is a useful approach to identify potential therapeutic targets. Knockdown of VRK1 sensitizes cells to other cancer treatments based on DNA damage such as ionizing radiation or doxorubicin by impairing the DNA damage response [46, 65]. Moreover, depletion of VRK1 inhibits cell proliferation [113, 128]. VRK1 has been identified as a potential target in a screening of synthetic lethal relationships in a massive siRNA screening [129]. These observations suggests that inhibitors of VRK1 can be of potential use in cancer treatments, by themselves or in combinations, by facilitating inhibition of proliferation and at the same time sensitizing cells to treatments based on DNA damage. In cancer treatment, many drugs are directed to the main driver as targets. However, cancer cells can be derailed if alternative pathways that impinge on basic processes of the tumor phenotype are targeted. These alternative targets will open a wide range of possibilities, as well as provide with alternatives to manage individual cases.

Kinases share a common structure in their catalytic kinase domain and are druggable proteins [130]. Therefore, the likelihood of cross inhibition with other kinases is very high and makes many kinase inhibitors promiscuous. In the human kinome, there are kinases that are isolated from other major branches, among which is VRK1. The VRK family has some structural differences, which makes its members susceptible of highly specific inhibition with no promiscuity as detected in kinase assays or by structural thermal shift upon binding to inhibitors [131, 132]. However, at this time, there is no specific inhibitor for VRK1. Testing kinases inhibitors that target the main kinome families did not detect any that has an effect in assays of VRK1 autophosphorylation and VRK1 phosphorylation of p53 or H3 [30]. This is due to the very high inhibitor concentrations needed because of their low affinity, which required doses in the micromolar range that have a very high risk of cross inhibition and of the high probability of high toxicity and side effects. The elimination of VRK1 causes a defective DDR that facilitates and increases the sensitivity to DNA damaging agents, such as ionizing radiation or doxorubicin [65]. Depletion of VRK1 sensitizes cells to these treatments because of defective DNA repair, and thus permits the use of lower doses of toxic drugs to achieve the same result. This is important, because this sensitization also occurs in non-dividing cells, and might be useful for targeting non-dividing cells within a tumor that later might cause a relapse. Moreover, the treatment with a lower dose of commonly used cancer drugs can contribute to a reduction of the toxicity associated with them.

Facilitating some degree of DNA damage in tumor cells can contribute to the generation of new antigens and facilitate the response to new therapies based on manipulation of the immune system, as supported by the evidence that tumors with an intrinsic higher genome instability are better responders to these new therapies [133].

In conclusion, the pharmacological targeting of VRK1 will impair p53-mediated responses, prevent cell cycle progression and proliferation, and sensitize cells to treatments based on DNA damage, such as ionizing radiation and some chemotherapeutic drugs. The consequence of therapeutically exploiting this target will be a better control of the tumor if the new drugs are selective regarding both its molecular target and the specific tumor cell.

## Abbreviations

VRK1: Vaccinia-related kinase 1
DSB: DNA double-strand break
DDR: DNA damage response
NBS1: Nijmegen breakage syndrome 1 (nibrin)
NHEJ: Non-homologous end-joining
53BP1: Tumor protein P53 binding protein 1
ATM: Ataxia-telangiectasia-mutated Ser/Thr kinase
